# Supplementary material for: Content-rich biological network constructed by mining PubMed abstracts
Source: BMC Bioinformatics. 2004 Oct 8;5:147. doi: 10.1186/1471-2105-5-147 (PMC528731; doi:10.1186/1471-2105-5-147)
Supplement: Additional File 5 — The original Chilibot query results of the term "long-term potentiation (LTP)" and 22 other terms, limiting the latest references analyzed to the years 1990, 1995, 2000, and 2004. [file 1471-2105-5-147-S5.bz2 › chilibotAdditionalFile5/ltp2004/html/PKC_NMDA.html]

 


 **PKC** and **NMDA** 
  
Found 237 abstracts in PubMed,  **30 abstracts were retrieved and analyzed**.  


---

 Search Google  |
 PDF files only 
|  EDU domain only 

---

**Interactive relationship** (e.g. stimulation, inhibition, etc)

- It appears that this negative regulation involves suppression of downstream signaling of  **NMDA**  receptor such as those mediated by  **PKC** .  Ref: 12710978 Neuroscience, 2003
- Here, we find that PKA and  **PKC**  phosphorylation sites flanking the RXR motif of the  **NMDA**  receptor NR1 subunit suppress ER retention and regulate receptor forward trafficking.  Ref: 14529714 Neuropharmacology, 2003
- Infusion of  **NMDA**  antagonist D AP5 prevented both the thermal hyperalgesia and the increase in  **PKC**  gamma expression in PTX treated rats.  Ref: 14768516 Acta Anaesthesiol Sin, 2003
- Coordinated PKA and  **PKC**  phosphorylation suppresses RXR mediated ER retention and regulates the surface delivery of  **NMDA**  receptors.  Ref: 14529714 Neuropharmacology, 2003
- Of the  **PKC**  inhibitor  **PKC**  I... through the recording electrode totally blocked clozapine s potentiating effect, indicating that a post synaptic expressed  **PKC**  is critically involved in the augmenting action of clozapine on  **NMDA**  evoked currents.  Ref: 12699785 Neuroscience, 2003
- Intracellular injection of the  **PKC**  inhibitor  **PKC**  I... through the recording electrode totally blocked clozapine s potentiating effect, indicating that a post synaptic expressed  **PKC**  is critically involved in the augmenting action of clozapine on  **NMDA**  evoked currents.  Ref: 12699785 Neuroscience, 2003
- PLC beta 1 TC mutants develop a loss of  **NMDA**  receptors in the stratum oriens of region CA1, apoptotic neuronal death, and reduced hippocampal  **PKC**  activity.  Ref: 12504592 Mol Cell Neurosci, 2002
- **NMDA**  also caused activation of  **PKC**  as evidenced by their phosphorylations.  Ref: 12950461 J Neurochem, 2003
- Intriguingly, the activity of protein kinase C  **PKC** , an enzyme regulated downstream of  **NMDA**  receptors, is increased in lyn mice.  Ref: 12710978 Neuroscience, 2003
- Epsilon  **PKC**  is required for the induction of tolerance by ischemic and  **NMDA**  mediated preconditioning in the organotypic hippocampal slice.  Ref: 12533598 J Neurosci, 2003
- These results demonstrate that BDNF functions at the spinal level to enhance synaptic efficacy in an  **NMDA**  receptor dependent manner and requires the action of the PLC  **PKC**  pathway.  Ref: 14622147 Eur J Neurosci, 2003
- Taken together, these results suggest that the  **PKC**  signal transduction pathway is critically involved in the facilitating action of clozapine on the  **NMDA**  induced responses in pyramidal cells of the mPFC.  Ref: 12699785 Neuroscience, 2003
- For this purpose, calcium i changes after  **NMDA**  receptor activation were monitored in primary cultures of embryonic rat hippocampal pyramidal neurons using microspectrofluorometry of the calcium sensitive indicator Fura 2 acetoxymethyl ester in the presence of sigma1 agonists and  **PKC**  inhibitors.  Ref: 12975497 J Pharmacol Exp Ther, 2003
- The fact that they mimicked those of MK801 suggests that presynaptic  **PKC**  may be involved in the  **NMDA**  receptor driven stabilization of developing retinal arbors.  Ref: 14750146 J Neurobiol, 2004
- Both tyrosine kinase and  **PKC** ... appear to be essential for EtOH withdrawal hyper responsiveness mediated by postsynaptic  **NMDA**  receptors in spinal cord motor neurons.  Ref: 12746225 Br J Pharmacol, 2003
- Together with previous studies, these results indicate that calcium influx elicited by active  **NMDA**  receptors and L VGCC triggers the Pyk2 Src signaling pathway mediated by  **PKC** , which aggravates cerebral ischemia lesions through up regulating the function of  **NMDA**  receptors after the onset of ischemia, and also could be regulated partly by CaM dependent kinases like CaMKII.  Ref: 14732460 Neurosci Lett, 2004

**Parallel relationship** (e.g. studied together, co-existance, homology, etc.)

- These results indicate that the translocation of the  **PKC**  caused by the calcium i elevation through  **NMDA**  receptors is concerned with the initial stage of error signal induced plasticity in the RA.  Ref: 12657904 Neuroreport, 2003
- These results indicate that ER retention of  **NMDA**  receptors is tightly regulated, and suggest that coordinated phosphorylation by PKA and  **PKC**  mediates release of receptors from the ER for subsequent traffic to synapses.  Ref: 14529714 Neuropharmacology, 2003
- Intrathecal administration at the T7 T9 spinal levels of an  **NMDA**  receptor antagonist D AP microg or a selective  **PKC**  inhibitor GF109203X, 0.15 microg significantly reversed the changes in exploratory activity when compared with the vehicle treated group of rats with experimental pancreatitis.  Ref: 14724052 Pharmacol Biochem Behav, 2004
- The present study therefore assessed whether the potentiating action of sigma1 agonists on the  **NMDA**  response observed in the hippocampus involves the regulation of calcium i and  **PKC** .  Ref: 12975497 J Pharmacol Exp Ther, 2003
- For comparison, we examined arbors treated with the  **NMDA**  blocker MK801 at half hour time lapse intervals, and detected the twofold rise in rates of branch addition and deletion previously reported in Xenopus larvae, but not the structural effect seen with the  **PKC**  inhibitors.  Ref: 14750146 J Neurobiol, 2004
- The increase of p JNK1& ; 2 was largely prevented by blockage of  **NMDA**  receptor a subtype of glutamate receptor or protein kinase C  **PKC** , and each blockage also largely prevented the cell death.  Ref: 12445686 Brain Res, 2002
- Lastly, to determine whether a protein kinase C  **PKC**  signaling system might be involved in the inhibition of  **NMDA**  stimulated 3H dopamine release, we tested the PKCbeta selective inhibitor 1 1 7 dimetheno 18H dibenzo i, o pyrrolo 1 diacyclohexadecine 1 0 19H dione, 8 dimethylamino methyl,, 1 1 hexahydro monomethanesulfonate 9Cl LY379196 against both progesterone and pregnenolone.  Ref: 12750428 J Pharmacol Exp Ther, 2003
- i.t. administration of N methyl D aspartate  **NMDA**  receptor antagonist, D 2 amino 5 phosponovaleric acid D AP5, glycine and protein kinase C  **PKC**  inhibitor chelerythrine attenuated the thermal hyperalgesia.  Ref: 14768516 Acta Anaesthesiol Sin, 2003
- We concluded that an intact  **PKC**  system was required for sigma1 agonist mediated regulation of  **NMDA**  stimulated 3H dopamine release from rat striatal slices.  Ref: 12490613 J Pharmacol Exp Ther, 2003
- Several lines of evidence suggest a role for protein kinase C  **PKC**  isoforms in  **NMDA**  excitotoxicity.  Ref: 12871585 J Neurochem, 2003
- Our results demonstrate that pancreatitis pain is the result of central pain processes that play a role in the amplification of responses to peripheral visceral input through  **NMDA**  receptor activation and  **PKC**  phosphorylation signaling pathways.  Ref: 14724052 Pharmacol Biochem Behav, 2004
- These results suggested that mainly downstream of  **NMDA**  receptor  **PKC**  pathway JNK1& ; 2 were activated, nuclear translocated and causally involved in the glutamate induced excitotoxicity, possibly through a nuclear elevation of p JNK1& ; 2.  Ref: 12445686 Brain Res, 2002
- Immunocytochemistry study showed at 15 min of glutamate exposure a whole cellular but mainly nuclear increase of p JNK1& ; 2, together with mild plasma decrease but large nuclear increase of JNK1& ; 2, all of which were also largely prevented by blockage of  **NMDA**  receptor or  **PKC** .  Ref: 12445686 Brain Res, 2002
- The main goals of the present study were to determine whether  **NMDA**  induces preconditioning via a calcium pathway and promotes translocation of the protein kinase C epsilon epsilonPKC isozyme and whether this  **PKC**  isozyme is key in the IPC signal transduction pathway.  Ref: 12533598 J Neurosci, 2003
- Bath administration of the  **PKC**  activator phorbol 12 myristate 13 acetate PMA... significantly enhanced the  **NMDA**  evoked inward current and electrically evoked excitatory postsynaptic currents.  Ref: 12699785 Neuroscience, 2003
- Restoration of spontaneous exploratory behaviors with an intrathecal  **NMDA**  receptor antagonist or a  **PKC**  inhibitor in rats with acute pancreatitis.  Ref: 14724052 Pharmacol Biochem Behav, 2004
